# Atomically resolved real-space imaging of hot electron dynamics

**DOI:** 10.1038/ncomms9365

**Published:** 2015-09-21

**Authors:** D. Lock, K. R. Rusimova, T. L. Pan, R. E. Palmer, P. A. Sloan

**Affiliations:** 1 Department of Physics, University of Bath, Bath, BA2 7AY, UK; 2 Nanoscale Physics Research Laboratory, School of Physics and Astronomy, University of Birmingham, Birmingham, B15 2TT, UK

## Abstract

The dynamics of hot electrons are central to understanding the properties of many electronic devices. But their ultra-short lifetime, typically 100 fs or less, and correspondingly short transport length-scale in the nanometre range constrain real-space investigations. Here we report variable temperature and voltage measurements of the nonlocal manipulation of adsorbed molecules on the Si(111)-7 × 7 surface in the scanning tunnelling microscope. The range of the nonlocal effect increases with temperature and, at constant temperature, is invariant over a wide range of electron energies. The measurements probe, in real space, the underlying hot electron dynamics on the 10 nm scale and are well described by a two-dimensional diffusive model with a single decay channel, consistent with 2-photon photo-emission (2PPE) measurements of the real time dynamics.

## Abstract (web summary)

Hot electrons—electrons with very high kinetic energies—are important in many processes but difficult to observe due to their short lifetimes. Here, the authors analyse STM based nonlocal manipulation of organic molecules on a surface, showing that the measurements probe hot electron dynamics in real space.

## Graphical abstract

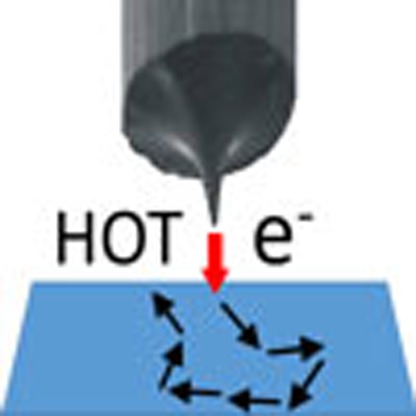

The dynamics of hot electrons, those with energies perhaps hundreds of times greater than the available thermal energy, is central to understanding the properties of many electronic devices. For example, they contribute to leakage currents across CMOS transistors and may fundamentally limit their downsizing^1^,^2^,^3^ and they are intrinsic to solar cells, where high energy photons generate hot electrons that consequently generate a photocurrent^4^,^5^,^6^. Hot electrons can also, for example, be induced through plasmonics^7^,^8^,^9^ and offer a route to instigate non-thermal chemical reactions^10^. Hot electron charge transport is thus the subject of major scientific research, both experimentally and theoretically^11^,^12^,^13^,^14^. But the experiments are constrained by the ultra-short lifetime of hot electrons, typically 100 fs or less, and hence their corresponding transport length-scale of a few nm (refs 15, 16). Direct pump–probe measurements with ultra-fast lasers typically provide little or no spatial information. Recently a scanning tunneling microscope (STM) coupled to terahertz radiation reported 0.5 ps and 2 nm resolution^17^. Here we employ the technique of STM-induced nonlocal atomic manipulation on an atomically precise surface to show that the nonlocal effect is a direct manifestation of hot electron dynamics. This allows us to observe directly and control the outcome of injecting hot electrons with atomic-scale spatial resolution.

Conventional atomic manipulation with the STM, whereby individual atoms and molecules are excited by the STM tunnel current, is constrained to the tip-surface tunnel junction 18,19,20,21,22,23,24,25. However, atomic manipulation can also occur some distance, ∼10 nm, from the tunnel junction. Such nonlocal manipulation can be considered as a three step process: (1) charge injection (electrons or holes) from the STM tip into the surface; (2) charge transport across the surface; and (3) electron (or hole) induced manipulation, for example, desorption or diffusion, of a remote adsorbate or substrate atom/molecule. Such nonlocal manipulation has now been reported across a range of systems, including metal^26^,^27^,^28^ and semiconductor^29^,^30^ surfaces, within chemical overlayers^31^ and on graphene^32^. In particular, several different systems based on the Si(111)-7 × 7 surface have shown similar nonlocal manipulation, suggesting a common transport mechanism: specifically, NO molecules^33^, C_60_ macromolecules^34^, chlorine atoms^35^ and even the silicon surface atoms themselves^36^. Our previous nonlocal study^37^ examined the injection step, here we probe the resulting hot electrons transport across the surface.

We show that the behaviour of the nonlocal atomic manipulation properties of small aromatic molecules on the Si(111)-7 × 7 surface in the STM exhibit a distinct temperature, as well as voltage (energy) dependence. Quantitative experimental results lead to a model of two-dimensional (2D) diffusion of hot electrons across the surface and within the back-bond state 2 eV above the Fermi level, consistent with ultra-fast pump–probe experiments on the same surface. We conclude that nonlocal STM manipulation provides real-space information on the hot electron transport at the surface and, by implication, their femtosecond electron dynamics.

## Results

### STM imaging of nonlocal manipulation

**Figure 1:**
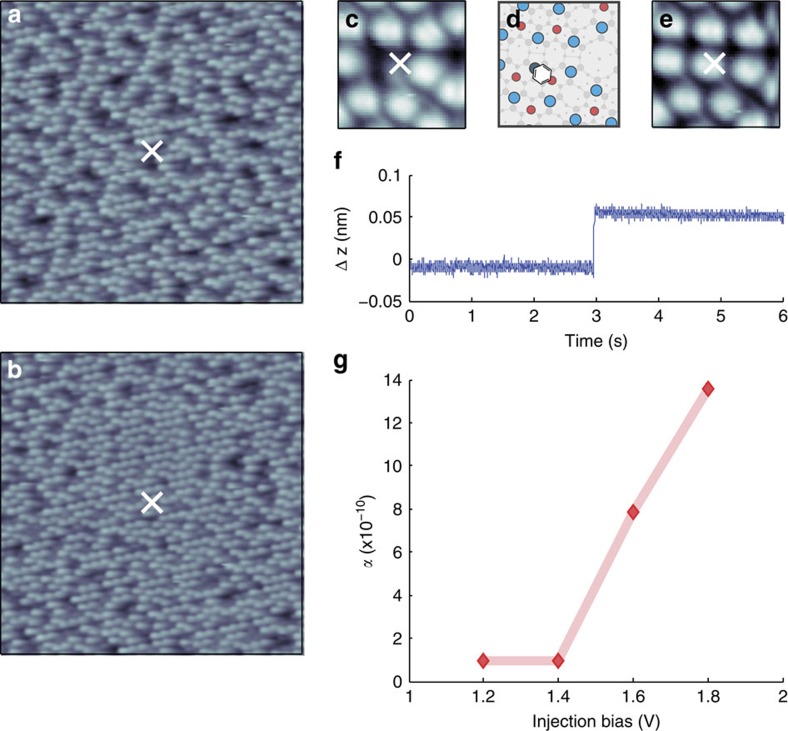
Nonlocal and local manipulation of toluene on the Si(111)-7 × 7 surface at room temperature. STM images (21 × 21 nm, +1 V, 100 pA) of toluene on Si(111)-7 × 7 taken before (**a**) and after (**b**) a charge injection (+2.7 V, 200 pA, 10 s) at location ‘X'. High resolution STM images (2.5 × 2.5 nm, +1 V, 100 pA) of toluene on Si(111)-7 × 7, taken before (**c**) and after (**e**) a charge injection (+1.6 V, 750 pA, 7 s) directly into the toluene molecule (dark spot) at location ‘X'. (**d**) Schematic diagram of a molecule binding to a restatom and adatom site. Time-trace (**f**) of the STM tip height above the molecule during the injection of C and E. (**g**) The probability per electron (*α*) as a function of injection voltage for a toluene molecule subject to manipulation (diffusion/desorption) by direct charge injection into the molecule.

Figure 1 shows a pair of room temperature STM images taken before (A) and after (B) injection of 2.7 eV electrons at the indicated central site. At the imaging bias voltage (+1 V) silicon adatoms image as bright spots and chemisorbed toluene molecules appear as dark spots and are unperturbed by the STM tunnel current 38. An area ∼15 nm in radius surrounding the hot-electron injection site is transformed, showing a marked reduction in the number of molecular adsorbates to reveal the underlying Si(111)-7 × 7 surface. We previously demonstrated this nonlocal manipulation to be a one-electron process^37^.

To disentangle the contributions of molecular and surface properties to this nonlocal manipulation, local manipulation experiments were performed. By positioning the STM directly over an adsorbate molecule (Fig. 1c,e) electrons were injected directly into a target molecule and a time-trace of the STM *z*-height recorded (Fig. 1f). The moment of displacement is indicated by the 0.05 nm step away from the surface as the molecule (dark spot) is removed to reveal the underlying silicon adatom (bright-spot). Figure 1d shows a schematic diagram of the bonding configuration of the lone toluene molecule. The exponential distribution of survival times from 107 individual time-traces gives the probability per injected electron *α* of inducing a manipulation event (see Methods for details). The voltage dependence of the probability per electron (Fig. 1g) displays a threshold energy of (1.4±0.1) eV. We conclude that any electron with energy below the 1.4 eV threshold that interacts with an adsorbate molecule, whether directly injected into a molecule from the STM tip or transported across the surface from a remote tip, will not induce manipulation. The molecules therefore function as a high-pass filter that discriminates against low-energy electrons and is only sensitive, unlike conventional surface conductivity measurements^39^,^40^, to high energy hot electrons.

The Si(111)-7 × 7 surface is known to be a conducting surface therefore it is perhaps not surprising that we find a spreading of the injected electrons across the surface^41^. However, even the best spatially resolved four-point-probe measurements that successfully isolate the surface conduction^39^ have a probe spacing of ∼10 μm, much larger than the <15 nm range of our nonlocal effect. Moreover, a conventional four-point-probe will measure low-energy transport across the surface as will a novel three-point-probe^40^, our nonlocal manipulation is sensitive to the hot electrons.

**Figure 2:**
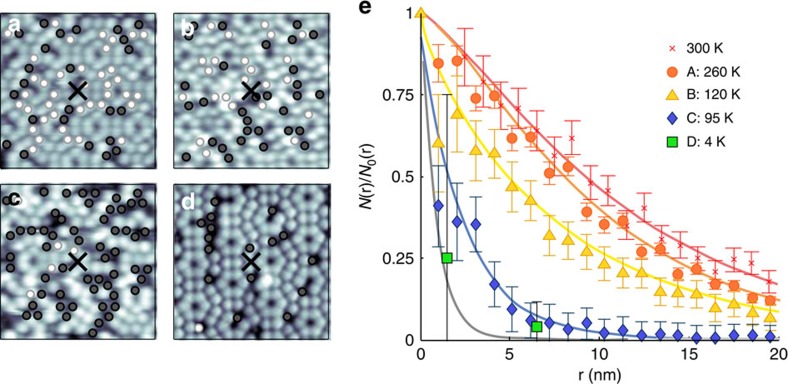
Nonlocal manipulation as a function of temperature. STM images (12 × 12 nm,+1 V, 100 pA) of chlorobenzene molecules on the Si(111)-7 × 7 surface following charge injection [+2.7 V, 800 pA, 80 s (**a**–**c**),+2.7 V, 100 pA, 160 s (**d**)] at the sites indicated by X: surface and tip at (**a**) 260 K, (**b**) 120 K, (**c**) 95 K and (**d**) 4 K. White circles indicate locations of molecules that are displaced due to the charge injection. Black circles indicate sites of molecules that are unmoved. (**e**) Radial distribution of displaced molecules as a function of the distance from the injection site at temperatures indicated. Each decay curve is the average of several (∼5) experiments. Lines are fits to the 2D diffusion, single decay channel transport model given by equation 1.

Figure 2 presents variable temperature STM images taken after electron injection into a surface at (A) 260 K, (B) 120 K, (C) 95 K and (D) 5 K. (The STM tip was always in thermal equilibrium with the sample.) Injecting into a colder surface results in a markedly reduced nonlocal effect. We quantitatively describe the nonlocal manipulation by counting the number of adsorbed molecules (versus radius) that move from their original position, that is molecules that have desorbed or diffused, within a 1-nm wide annulus at a certain radius. An automated computer programme 38 identifies the location of every dark-spot (that is, molecule) in images before and after injection within each annulus. We use this information to plot the ratio of the number of dark spots displaced (desorbed or diffused) from their original position *N*(*r*) (that is, the number of molecules manipulated) to the original number of dark spots *N*_0_(*r*). (We assume the same nonlocal mechanism drives both desorption and diffusion as both share the same initial bond-breaking step typical for a chemisorbed system.) Figure 2e presents curves showing the distance dependence of the ratio *N*(*r*)/*N*_0_(*r*) at five temperatures. The probability and range of nonlocal manipulation clearly increase as the temperature rises.

### Quantifying nonlocal manipulation through diffusive transport

The temperature dependence suggests that the injected electrons must, to some extent, thermalize yet remain above the 1.4 eV molecular energy threshold at all temperatures, that is, they are still hot electrons. Furthermore, the reduction of the nonlocal range rules out a simple ballistic transport mechanism, since this transport mode would be expected to lead to a longer range at lower temperature as the population of phonons available to scatter the electron is reduced at low temperature. Thus, we model the hot-electron dynamics with a diffusive transport model. We restrict the model to two dimensions, since we are only sensitive to the electrons at the surface. We use a single isotropic diffusion coefficient *D*, as we find no azimuthal angular dependence to the nonlocal manipulation. To account for the finite range of nonlocal manipulation we include a single decay channel with lifetime *τ* (see Methods section for details). The resulting mathematical model gives


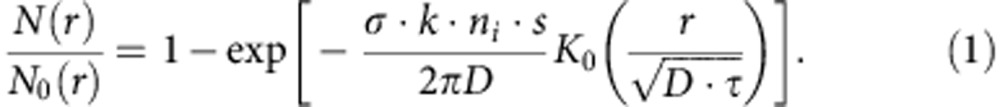


Here *n*_*i*_ is the total number of electrons injected during the voltage pulse, *s* is the (unknown) factor describing the fraction of the injected electrons initially captured into the 2D state (we set *s* to unity), *σ* is the cross-section for an electron that interacts with a molecule (analogous to gas-phase electron scattering) and *k* is the probability per unit time of an electron inducing a manipulation event once it reaches a molecule. Finally, *K*_0_ is a modified Bessel function of the second kind. Figure 2e shows the good fit of this model to the experimental data at the five temperatures shown. The surface charge diffusion model conforms to the observed insensitivity of the nonlocal manipulation to the type of dopant, n-type or p-type, and the dopant level from 30 to 0.1 Ω cm, since the surface electronic properties of Si(111)-7 × 7 are independent of those of the bulk (except at extreme doping levels) due to the pinning of the Fermi level by the surface dangling-bond state. Crucially the transport properties are regulated by a single parameter, the diffusion length 
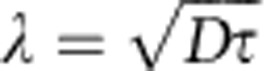
.

**Figure 3:**
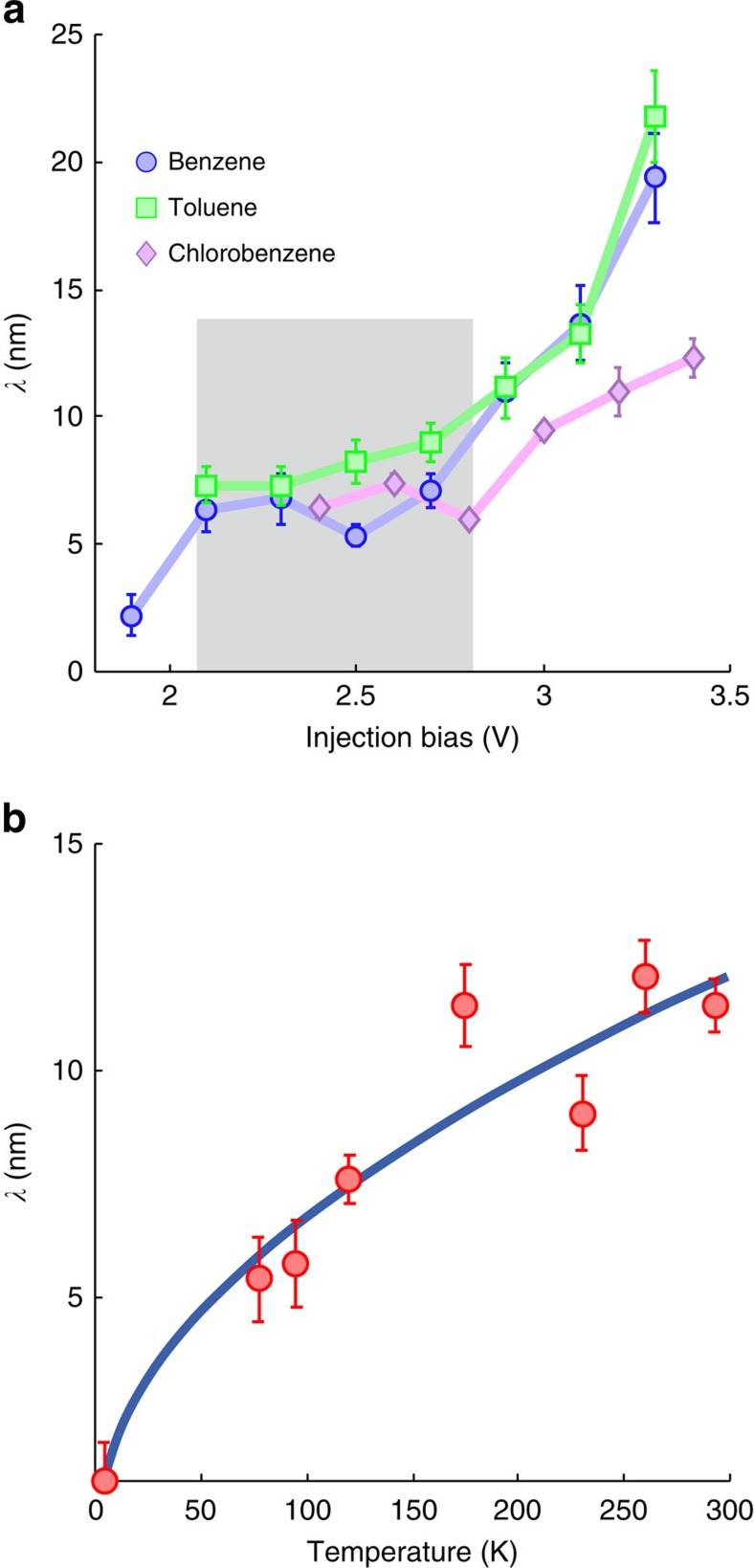
Range, *λ*, of nonlocal manipulation versus *V* and *T*. (**a**) Voltage dependence of the nonlocal manipulation range *λ* at room temperature (random injection site) for: benzene, blue circles; toluene, green squares; chlorobenzene, purple diamonds. Grey region highlights a region of (near) constant range. (**b**) Temperature dependence of the range *λ* for injection into corner-hole sites at +2.7 V for chlorobenzene molecules on Si(111)-7 × 7. Solid blue line is a fit to data (see Discussion for details) with *T*_*e*_∝*T*^1.5±0.2^.

Figure 3a plots the voltage dependence of the nonlocal range *λ* at room temperature for three similar adsorbate molecules: benzene, toluene and chlorobenzene. For all three molecules we find a manipulation onset at 2.0 eV, followed by a relatively constant plateau region of *λ* up to ∼2.8 eV (indicated by the grey region) followed by a rise in *λ*. Within the plateau region (2.0–2.8 eV) it appears that no matter what the injection energy, electrons are transported across the surface in almost identical fashion. This implies that before undergoing surface transport any electron injected within this energy window attains some common transport dependent property. Similar energy independent behaviour was reported for laser excitation of surface-state electrons of the Si(111)-7 × 7 surface.

## Discussion

In 2-photon-photo-emission (2PPE) an initial (pump) laser pulse excites electrons within a surface and a second (probe) pulse creates photoelectrons that are energy analysed. Such 2PPE experiment have reported energy^42^,^43^ and time-resolved measurements^44^ of the surface electronic states of Si(111)-7 × 7. For a range of pump energies, the probe pulse was found to generate photoelectrons from a common intermediate state with energy of (+1.94±0.15) eV (refs 42, 44) above *E*_F_. Between the pump and probe (<150 fs) the initially highly excited electron relaxes to a common energy state at +1.94 eV above *E*_F_ before photo-emission. By careful consideration of pump and probe polarizations, adsorption coefficients and energetics, and the symmetry of the initial state, the +1.94 eV state was identified as the unfilled back-bond surface-state *U*_2_ (ref. 42). The *U*_2_ state has been reported for a wide range of energies from 1.6 to 1.9 eV (ref. 42) and has been observed in numerous STM and ultraviolet photoelectron spectroscopy experiments^37^,^45^,^46^. Such a process, the ultra-fast relaxation on a state at 1.94 eV, would explain the voltage independence of the nonlocal range *λ* for injections between +2.0 and ∼2.8 V if, before the injected electron begins its diffusive transport, it has already relaxed down to the 1.94 eV state attaining a common energy and hence common transport dynamics.

At higher pump energies a new intermediary bulk-state arises in the 2PPE experiments at (3.45±0.15) eV above *E*_F_. The increase in our transport range between 2.8 eV and our maximum energy of 3.4 eV may be explained with the opening of a new diffusive route with longer range transport and much higher manipulation probability (not shown) through this new channel. Furthermore, this bulk-like transport channel may well also account for the insensitivity of nonlocal manipulation behaviour to grain boundaries and atomic steps for 3.3eV electron injections^37^,^47^.

Time-resolved 2PPE experiments^44^ of the 1.94 eV derived peak (though attributed to the bulk L-valley of silicon) show that between pump and probe pulses there is an ultrafast, <40 fs, convergence of the initially excited electrons onto an electronic resonance at 1.94 eV above the Fermi level. Furthermore, the time-dependent 2PPE signal of the electrons photoemitted from the 1.94 eV intermediate state was well described by a diffusion process coupled to a single decay channel; the lifetime for the hot electrons in the intermediate state was determined as (180±20) fs and the state to which electrons decay lay at +0.5 eV above the Fermi level. In our STM experiment, electrons with energy of +0.5 eV will be unable to induce a nonlocal manipulation event, as they will lie well below the 1.4eV manipulation energy threshold. On the basis of our measured length-scale of (7±1) nm and the 2PPE derived lifetime of (180±20) fs, we compute a diffusion coefficient at room temperature of (3±2) cm^2^ s^−1^. Our value of *D* is similar to, but smaller than, simulations of conduction band electrons at a silicon surface of 18 cm^2^  s^−1^ (ref. 48), that used in ultrafast measurements of free carriers in bulk silicon 20 cm^2^ s^−1^ (ref. 49) and for that found for carrier diffusion in single silicon core-shell nanowires of 35 cm^2^ s^−1^ (ref. 50).

Figure 3b presents the temperature dependence of the nonlocal length-scale *λ*. As the lattice temperature increases so does the nonlocal manipulation range. This further conforms to the diffusive transport model which, unlike ballistic transport, should increase with increasing temperature. The relationship also demonstrates the connection between the temperature of the crystal lattice and the measured electron dynamics.

Hot electrons undergo several scattering processes to bring them into eventual thermal equilibrium with a crystal lattice. Typically carrier–carrier and carrier–phonon scattering brings a population of hot carriers into a Fermi–Dirac distribution with some characteristic hot electron temperature *T*_*e*_ within 10 s of fs (refs 43, 51, 52). We inject at currents of ∼100 pA and therefore have, on average, one-electron injected every 1 ns. Given the 180 fs lifetime of the electrons within the 2.0 eV state, we rule out carrier–carrier scattering. Further carrier–phonon scattering brings the mean energy of the electron distribution down to ultimately thermalize with the lattice. In our case, this carrier–phonon scattering appears to occur within the high-lying 2.0 eV state since we have a temperature dependence of the nonlocal range. Subsequent scattering, here in a time of 180 fs, removes the electron from this state to lower lying states. There is therefore a competition between electron–phonon scattering thermalizing the hot-electron within the 2.0 eV state and scattering that electron into a lower lying state. This will result in a dynamic quasi-equilibrated electron distribution within the 2.0 eV state.

To gain an insight into this connection we present a simple model of the temperature dependence of the nonlocal effect in terms of a 2D random walk. The RMS of a 2D random walk can be equated to 2D diffusion by 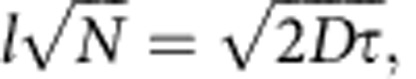 where *l* is the random walk step length and *N* the number of steps. Hence our length-scale 
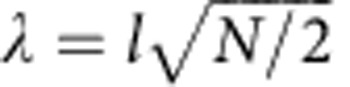
. We set the time between steps (scattering events) as *τ*_*s*_ then, 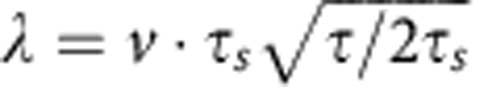. The decay mechanism out of the +2.0 eV state is by electron–phonon scattering^44^ giving *τ*=*Aτ*_ep_, where *A* is a constant that describes the probability of a scattering event relaxing the electron down to the dangling-bond state^53^ and *τ*_*ep*_ is the time between electron–phonon scattering events.

The temperature dependence of the rate of electron–phonon scattering varies as 2*n*_BE_(*T*)+1 where *n*_BE_(*T*) is the Bose–Einstein distribution for a particular phonon mode^53^, hence *τ*_ep_ ∝1/[2*n*_BE_(*T*)+1]. The 2.0 eV state is identified as lying at the silicon back-bond sites. At this back-bond site the phonon mode with the greatest spectral power has an energy of 53 meV (ref. 54). We assume the same scattering event that causes the electron to decay out of the state is also responsible for the 2D random walk giving *τ*_s_∝*τ*_ep_ and therefore 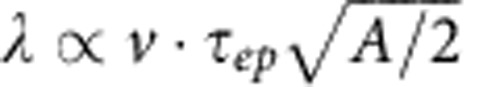. Given the electron has reached a quasi-equilibrated distribution with effective temperature *T*_e_ we can write, 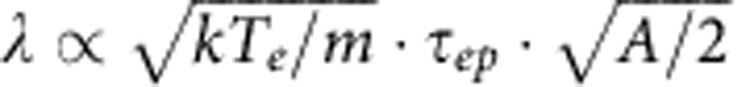. The temperature dependence of *λ* should vary as the temperature dependent terms 
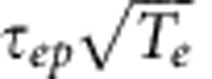
. Finally we simply set the power law relationship *T*_e_∝*T*^*n*^. The curve fitted in Fig. 3b, *λ*∝*T*^*n*^/[2*n*_BE_(*T*)+1], gives a value of *n*=1.5±0.2 and demonstrates the lattice temperature dependence of the hot-electron transport. Further analysis would need a more detailed theoretical framework.

## Methods

### Experimental

Room temperature STM images were obtained using an Omicron UHV STM-1 instrument with RHK SPM1000 controller for the nonlocal manipulation and with a Nanonis control system with customized software for the local manipulation work. Low temperature nonlocal measurements were performed using an Omicron UHV LT-STM with Nanonis electronics. All images were obtained in ‘passive'^38^ constant-current mode with a tunnelling current of typically 100 pA and a sample bias voltage of +1 V. STM tips were electrochemically etched from 0.25 mm tungsten wire in 2 M NaOH solution. Insulating tungsten-oxide was removed from the tips by resistive heating in high vacuum. Reproducible and clean Si(111)-7 × 7 surfaces were prepared using an automated computer controlled system. See ref. 37 for details of sample and gas preparation. The voltage dependent measurement used an n-type crystal with 30–0.1 Ω cm resistivity. The temperature dependent measurements used a p-type crystal with resistivity 0.01 Ω cm to enable STM imaging down to 4 K. A suite of in house written computer programs were used to determine the position of all molecules in the STM images before and after injection. A typical 65 nm × 65 nm image contained between 500 and 1,000 molecules for analysis with a molecular detection error rate of <1%. Thermally activated displacement was accounted for in the same manner as ref. 38. For each voltage 15 sets of before and after injection images were typically analysed, for each temperature five sets of before and after injection images were typically analysed. All errors quoted are one s.d.

All temperature dependent injections were performed into the corner-hole location of the Si(111)-7 × 7 surface. By contrast, the voltage dependent measurements had random injection sites. At the crossover energy ∼2.7 eV between transport through the *U*_2_ state and through the bulk-like states, we find a slight injection site dependence of the measured range *λ*. We attribute this to the site-specific energy landscape of the Si(111)-7 × 7 reconstruction which manifests itself in slightly different intensities of the surface states at each site. Thus, at a particular injection energy there is a site-dependence of the fraction of the tunnelling current injected into the lower or the upper manipulation state. At the crossover region, where both states are present on each site, we therefore measure an average length-scale *λ* weighted by the intensity of the transport state at the injection site. At the corner-hole site there is a higher intensity (than the surface averaged) of the higher lying state and hence the slightly longer manipulation range (Fig. 3b) of (11±1) nm than the range averaged over sites of (7±1) nm (Fig. 3a).

### Local manipulation

We work at room temperature and hence have more contamination issues than at lower temperature due to higher background pressures. Coupled to the fact that contamination on the Si(111)7 × 7 surface images in a near identical fashion to our adsorbate molecule, ‘dark-spots' on the Si(111)7 × 7 surface, non-reactive contaminants could be included in our local experiments and may skew our results. Here we present a slightly different method of extracting the probability per electron from local injection experiments than the standard recipe. This allows us to remove any bias in our results due to such background contamination.

The single-molecule experiments were performed with a Nanonis SPM control system, which was programmed to find and inject into a pre-selected molecular adsorption position. Stability during the injection was ensured by bespoke drift-compensation software, enhanced by a ‘feature locking' routine prior to injection. The time taken for the target molecule to be manipulated (desorbed or displaced) was simply read off a time-trace of the *z*-height of the tip during the injection (see Fig. 1f). Molecular manipulation was subsequently confirmed by comparison of the passive scans taken before and after an injection. It has been previously demonstrated that molecules adsorbed to the faulted middle adatom sites of the Si(111)-7 × 7 surface displace more readily than those adsorbed to other positions^37^. Therefore, we selected to inject only into molecules located at faulted middle positions. Given a first order rate dependence, the time-dependent probability of a molecule remaining in its initial position is


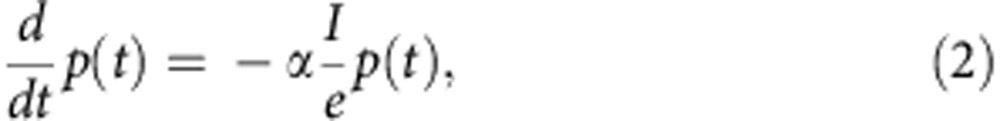


where α is the probability per electron of inducing a displacement of the molecule, *I* is the tunnelling current and *e* is the absolute charge on an electron. We solve with initial conditions *p*(0)=1 to get


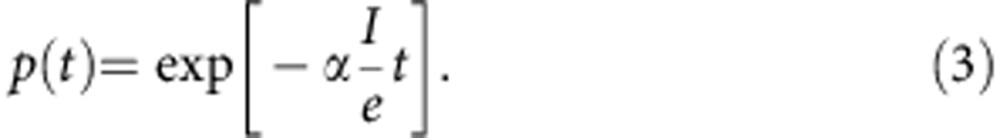


Experimentally we measure a population of individual molecules *N*_0_ and from aggregating the individual time-traces can construct the time-dependent population *N*(*t*) so that *N*(*t*)/*N*_0_=*p*(*t*). The value of *N*_0_ will include any ‘black-spot' contaminants that we have inadvertently included in our experiment and analysis. Even at low levels these can unduly skew the resulting analysis. To alleviate this problem we recast the analysis in terms of the number of molecules that are manipulated (rather than the number that remain in place). We define *G*(*t*) to be the time-dependent number of molecules that have been manipulated,


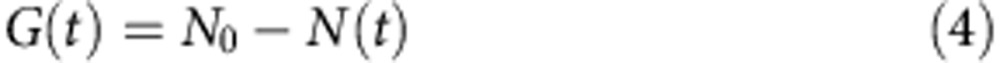



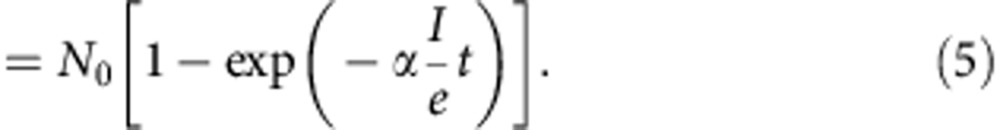


If we take the time for, say, the *N*th molecule of an initial population of *N*_0_ to be displaced as *t*_*N*_ then


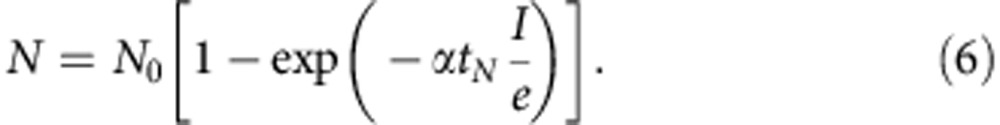


and hence,


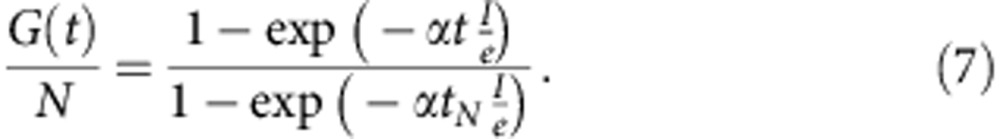


**Figure 4:**
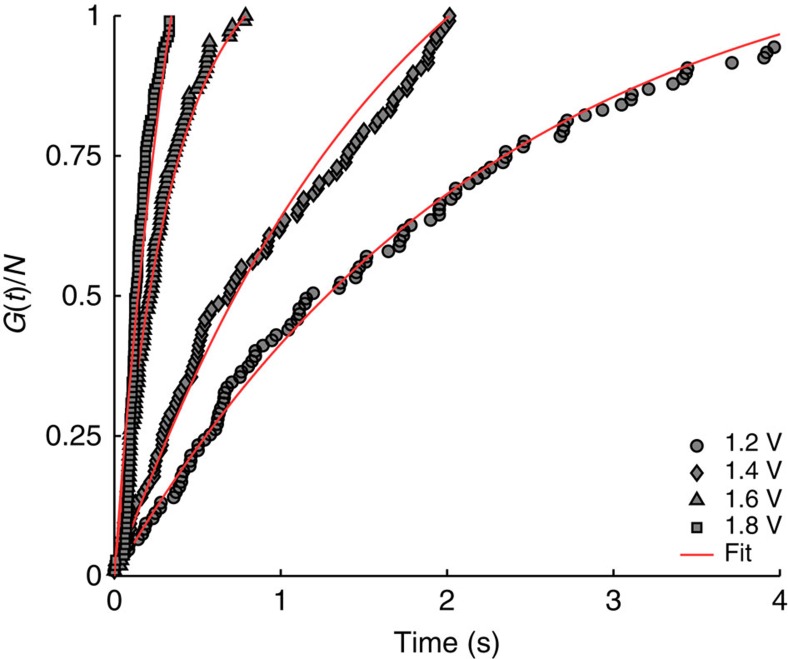
Number of manipulated molecules as a function of time for four injection bias voltages: circles, 1.2 V; diamonds, 1.4 V, triangles, 1.6 V and squares, 1.8 V. All injections are with 750 pA and *z*-feedback loop active. Fit details are given in the Methods section and fit parameters presented in Fig. 1f.

This effectively allows us to remove the initial number of dark-spots *N*_0_ from our analysis and leaves us with the single fitting parameter *α*. We compute the best fit to all our data for a range of *N* values and find that there is a large range of *N* that all lead to the same fitted value of *α*. Going beyond this range begins to add in contaminant effects with a resulting skew of the fitted *α* values. Figure 4 shows, a plot of this ratio (using *N*=107) and the appropriate mathematical fit with single fitting parameter *α*, for four bias voltages: 1.2, 1.4, 1.6 and 1.8 V (all with 50pA injection current). The results are presented in Fig. 1g. We note that the displacement at 1.2 V is higher than that we would expect based on thermal displacement^38^. It may be that the close proximity of the tip lowers the barrier between the chemisorbed and physisorbed state leading to this slightly enhanced thermally activated displacement.

### Nonlocal probability of manipulation of a single molecule by a single electron

We generically write the rate of change of the probability *p*(*r*) of a molecule retaining its original position at a distance *r* from the charge injection as the first order rate equation


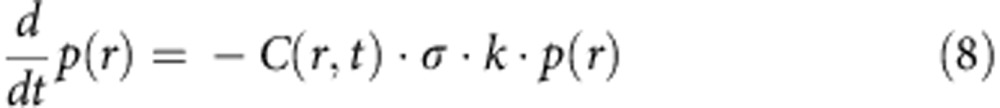


where *C*(*r*,*t*) is the time-dependent probability per unit area of finding an electron at that radial distance, *σ* is the cross-section of the molecule (analogous with gas-phase electron scattering) and *k* is the probability per unit time of an electron causing desorption. The factor *C*(*r*, *t*) is dependent on the transport model and we will describe a 2D-diffusive model in the next section.

Solving with initial conditions (*p*(*r*)=1 at *t*=0) gives


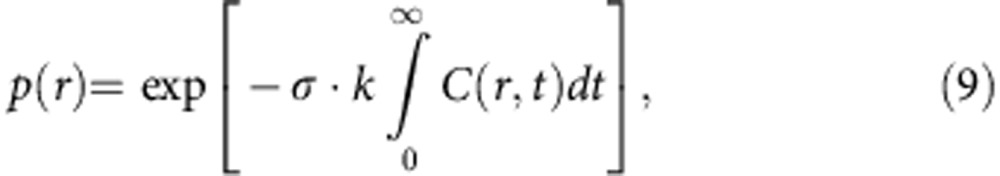


where the integral runs over the whole lifetime of the single electron during the manipulation. This is of the order 100 fs. During the several seconds injection we have many injected electrons, each of which may induce desorption. Hence, we write the probability of a molecule desorbing *P*(*r*) at a distance *r* after an injection containing *n*_*i*_ electrons as


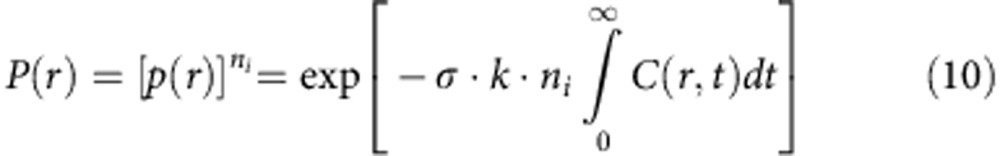


Experimentally we measure the number of molecules that leave their original location *N*(*r*) due to an injection of current *I* and duration *t*_*i*_, and the original number of molecules *N*_0_(*r*) within an annulus at radius *r* hence


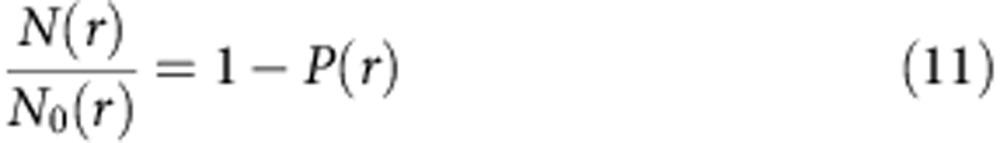



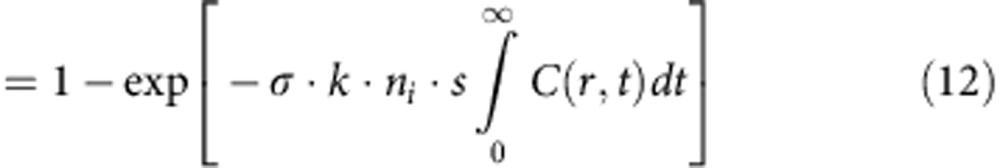


where *n*_*i*_=*It*_*i*_/*e* and *s* is a (unknown) factor describing the fraction of the injected electrons that are initially captured into the 2D state.

We note that even with a modicum of contamination this gives a robust fit (since we have ∼1000 data points per injection experiment) and purposely do not drive all molecules to be manipulated. We aim for half the molecules to be manipulated at half the distance between the injection site and the boundary of the before and after STM images.

### 2D-diffusive transport model

The normalized analytical time and radial distance dependent solution for 2D diffusion from an instantaneous point source at *r*=0 and *t*=0 in an unbounded domain assuming isotropic diffusion coefficient *D* is


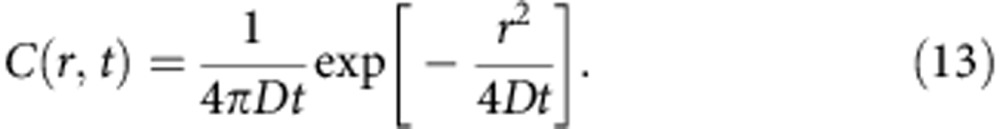


We add a single decay term to give


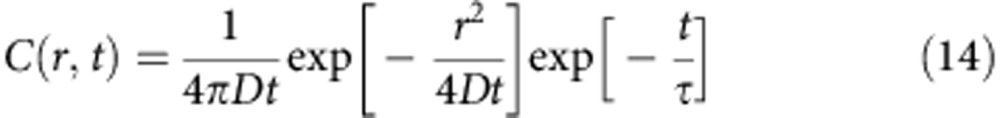


with a lifetime *τ*. We typically use a tunnel current of 100 pA, so on average electrons arrive every 1 ns. Since this is much longer than the lifetime of a hot electron (∼100 fs) we can assume there is only one electron in the surface at a time (consistent with a one-electron process). Hence, the time integrated total charge per unit area at a distance *r* for an initial single electron at *r*=0 is


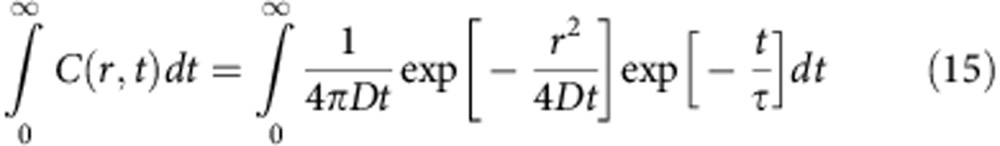



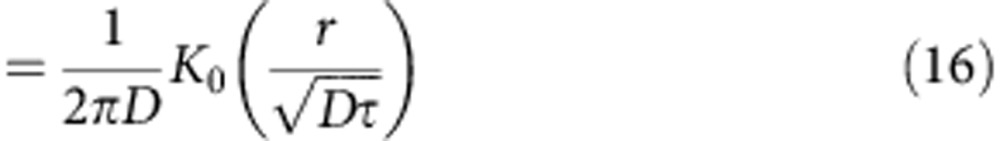


where 
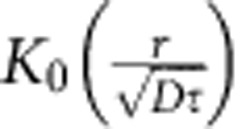
 is a modified Bessel function of the second kind with argument 
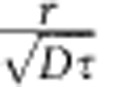
 and 
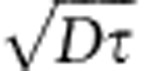
 is nothing more than the diffusion length which we write as *λ*. Finally we can write that


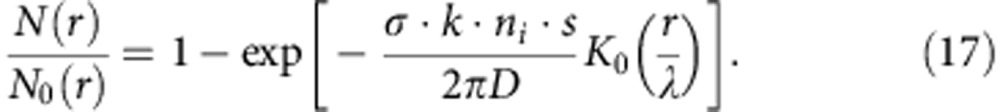


Therefore the length-scale of the nonlocal effect, in this model, is only dependent on the transport of the hot electrons, specifically their diffusion coefficient and their lifetime. For a diffusion length of ∼10 nm, the Bessel function mimics an exponential decay between ∼6 and ∼20 nm in agreement with our initial model given in ref. 37.

## Additional information

**Accession codes**: All data supporting this study are openly available from the University of Bath data archive at http://dx.doi.org/10.15125/BATH-00126.

**How to cite this article**: Lock, D. *et al*. Atomically resolved real-space imaging of hot electron dynamics. *Nat. Commun.* 6:8365 doi: 10.1038/ncomms9365 (2015).
